# Common Variants Near ZIC1 and ZIC4 in Autopsy-Confirmed Multiple System Atrophy

**DOI:** 10.1002/mds.29164

**Published:** 2022-08-23

**Authors:** Franziska Hopfner, Anja K. Tietz, Viktoria C. Ruf, Owen A. Ross, Shunsuke Koga, Dennis Dickson, Adriano Aguzzi, Johannes Attems, Thomas Beach, Allison Beller, William P. Cheshire, Vivianna van Deerlin, Paula Desplats, Günther Deuschl, Charles Duyckaerts, David Ellinghaus, Valentin Evsyukov, Margaret Ellen Flanagan, Andre Franke, Matthew P. Frosch, Marla Gearing, Ellen Gelpi, Jay A. van Gerpen, Bernardino Ghetti, Jonathan D. Glass, Lea T. Grinberg, Glenda Halliday, Ingo Helbig, Matthias Höllerhage, Inge Huitinga, David John Irwin, Dirk C. Keene, Gabor G. Kovacs, Edward B. Lee, Johannes Levin, Maria J. Martí, Ian Mackenzie, Ian McKeith, Catriona Mclean, Brit Mollenhauer, Manuela Neumann, Kathy L. Newell, Alex Pantelyat, Manuela Pendziwiat, Annette Peters, Laura Molina Porcel, Alberto Rabano, Radoslav Matěj, Alex Rajput, Ali Rajput, Regina Reimann, William K. Scott, William Seeley, Sashika Selvackadunco, Tanya Simuni, Christine Stadelmann, Per Svenningsson, Alan Thomas, Claudia Trenkwalder, Claire Troakes, John Q. Trojanowski, Ryan J. Uitti, Charles L. White, Zbigniew K. Wszolek, Tao Xie, Teresa Ximelis, Yebenes Justo, Alzheimer’s Disease Genetics Consortium, Ulrich Müller, Gerard D. Schellenberg, Jochen Herms, Gregor Kuhlenbäumer, Günter Höglinger

**Affiliations:** 1 Department of Neurology Hannover Medical School, Hannover, Germany; 2 Department of Neurology, Kiel University, Kiel, Germany; 3 Center for Neuropathology and Prion Research, Ludwig-Maximilians-Universität, Munich, Germany; 4 Department of Neuroscience, Mayo Clinic, Jacksonville, Florida, USA; 5 Department of Clinical Genomics, Mayo Clinic, Jacksonville, Florida, USA; 6Department of Neuroscience (Neuropathology), Mayo Clinic, Jacksonville, Florida, USA; 7 Institute of Neuropathology, University Hospital Zürich, Zürich, Switzerland; 8 Translational and Clinical Research Institute, Newcastle University, Newcastle upon Tyne, United Kingdom; 9 Banner Sun Health Research Institute, Sun City, Arizona, USA; 10 Department of Pathology, University of Washington, Seattle, Washington, USA; 11 Department of Neurology, Mayo Clinic, Jacksonville, Florida, USA; 12 Department of Pathology and Laboratory Medicine, Penn Neurodegeneration Genomics Center, Perelman School of Medicine, University of Pennsylvania, Philadelphia, Pennsylvania, USA; 13 Department of Neurosciences, School of Medicine University of California San Diego, La Jolla, California, USA; 14 Department of Pathology, School of Medicine University of California San Diego, La Jolla, California, USA; 15 Institut du Cerveau, UMR 7225, Sorbonne Université, Paris Brain Institute-ICM, CNRS, AP-HP, Hôpital de la Pitié Salpêtrière, Inserm U1127 DMU Neurosciences, Paris, France; 16 Brainbank NeuroCEB Neuropathology Network: Plateforme de Ressources Biologiques, Hôpital de La Pitié-Salpêtrière, Bâtiment Roger Baillet, Paris Cedex, France; 17 Institute of Clinical Molecular Biology, Christian-Albrechts-University of Kiel & University Hospital Schleswig-Holstein, Kiel, Germany; 18 Mesulam Center for Cognitive Neurology and Alzheimer’s Disease Northwestern University Feinberg School of Medicine, Chicago, Illinois, USA; 19 Department of Pathology, Northwestern University, Chicago, Illinois, USA; 20 Department of Neurology, Massachusetts General Hospital, Boston, Massachusetts, USA; 21 Harvard Medical School, Boston, Massachusetts, USA; 22 Department of Pathology, Massachusetts General Hospital, Boston, Massachusetts, USA; 23 Departments of Pathology and Laboratory Medicine and Neurology, Emory University School of Medicine, Atlanta, Georgia, USA; 24 Division of Neuropathology and Neurochemistry, Department of Neurology, Medical University of Vienna, Vienna, Austria; 25 Medical University of Vienna, Austrian Reference Center for Human Prion Diseases (OERPE), Vienna, Austria; 26 Department of Pathology and Laboratory Medicine, Indiana University School of Medicine, Indianapolis, Indiana, USA; 27 Department of Neurology, Emory University, Atlanta, Georgia, USA; 28 Memory and Aging Center, Weill Institute for Neurosciences, Department of Neurology, University of California San Francisco, San Francisco, California, USA; 29 Global Health Institute, University of California, San Francisco, California, USA; 30 Department of Pathology, University of California, San Francisco, California, USA; 31 Department of Pathology, University of Sao Paulo Medical School, Sao Paulo, Brazil; 32 The University of Sydney, School of Medical Sciences, and Brain & Mind Centre, Sydney, New South Wales, Australia; 33 Department of Biomedical Informatics, Columbia University Irving Medical Center, New York, New York, USA; 34 Department of Biomedical and Health Informatics, Children’s Hospital of Philadelphia, Philadelphia, Pennsylvania, USA; 35 Division of Neurology, Children’s Hospital of Philadelphia, Philadelphia, Pennsylvania, USA; 36 The Epilepsy NeuroGenetics Initiative (ENGIN), Children’s Hospital of Philadelphia, Philadelphia, Pennsylvania, USA; 37 Department of Neurology, Perelman School of Medicine, University of Pennsylvania, Philadelphia, Pennsylvania, USA; 38 Department of Neuroimmunology, Netherlands Institute for Neuroscience, Amsterdam, the Netherlands; 39 Brain Plasticity Group, Swammerdam Institute for Life Sciences, University of Amsterdam, Amsterdam, the Netherlands; 40 Department of Neurology, University of Pennsylvania Perelman School of Medicine, Philadelphia, Pennsylvania, USA; 41 Department of Laboratory Medicine and Pathobiology and Tanz Centre for Research in Neurodegenerative Diseases, University of Toronto, Toronto, Ontario, Canada; 42 Laboratory Medicine Program and Krembil Brain Institute, University Health Network, Toronto, Ontario, Canada; 43 Department of Pathology and Laboratory Medicine, Translational Neuropathology Research Laboratory Perelman School of Medicine, Philadelphia, Pennsylvania, USA; 44 DZNE – German Center for Neurodegenerative Diseases, Munich, Germany; 45 Munich Cluster for Systems Neurology (SyNergy), Munich, Germany; 46 Department of Neurology, Ludwig-Maximilians-Universität München, Munich, Germany; 47 Parkinson’s Disease and Movement Disorders Unit, Department of Neurology, Hospital Clinic of Barcelona, Barcelona, Spain; 48 Institut de Neurociències, Maeztu Center, University of Barcelona, Barcelona, Spain; 49 Institut d’Investigacions Biomèdiques August Pi i Sunyer (IDIBAPS), Barcelona, Spain; 50 Centro de Investigacion Biomédica en Red sobre Enfermedades Neurodegenerativas (CIBERNED), Barcelona, Spain; 51 Department of Pathology, University of British Columbia, Vancouver, British Columbia, Canada; 52 Department of Pathology, Vancouver General Hospital, Vancouver, British Columbia, Canada; 53 Department of Anatomical Pathology, Alfred Health, Melbourne, Victoria, Australia; 54 Paracelsus-Elena-Klinik, Kassel, Germany; 55 Department of Neurology, University Medical Center Goettingen, Gottingen, Germany; 56 Molecular Neuropathology of Neurodegenerative Diseases, German Center for Neurodegenerative Diseases, Tübingen, Germany; 57 Department of Neuropathology, University Hospital of Tübingen, Tübingen, Germany; 58 Department of Pathology and Laboratory Medicine, Indiana University School of Medicine, Indianapolis, Indiana, USA; 59 Department of Neurology, School of Medicine, Johns Hopkins University, Baltimore, Maryland, USA; 60 Department of Neuropediatrics, Children’s Hospital, University Medical Center Schleswig-Holstein, University of Kiel, Kiel, Germany; 61 Institute of Clinical Molecular Biology, Christian-Albrechts-University of Kiel, Kiel, Germany; 62 Institute of Epidemiology, Helmholtz Zentrum München, German Research Center for Environmental Health, Neuherberg, Germany; 63 Neurology Department, Hospital Clinic, IDIBAPS, Barcelona, Spain; 64 Neuropathology Department, CIEN Foundation, Alzheimer’s Centre Queen Sofía Foundation, Madrid, Spain; 65 Department of Pathology, 3rd Faculty of Medicine, Charles University, University Hospital Kralovske Vinohrady, Prague, Czech Republic; 66 Department of Pathology and Molecular Medicine, 3rd Faculty of Medicine, Charles University, Thomayer University Hospital, Prague, Czech Republic; 67 Division of Neurology, Royal University Hospital, University of Saskatchewan, Saskatoon, Saskatchewan, Canada; 68 Saskatchewan Movement Disorders Program, Saskatchewan Health Authority/University of Saskatchewan, Saskatoon, Saskatchewan, Canada; 69 John P. Hussman Institute for Human Genomics and Dr. John T. Macdonald Department of Human Genetics, University of Miami, Miller School of Medicine, Miami, Florida, USA; 70 Basic and Clinical Neuroscience Department, Institute of Psychiatry, Psychology and Neuroscience, King’s College London, London, United Kingdom; 71 Department of Neurology, Northwestern University Feinberg School of Medicine, Chicago, Illinois, USA; 72 Institute for Neuropathology, University Medical Centre Göttingen, Göttingen, Germany; 73 Section of Neurology, Department of Clinical Neuroscience, Karolinska Institutet, Stockholm, Sweden; 74 Department of Neurosurgery, University Medical Center Goettingen, Goettingen, Germany; 75 Department of Pathology and Laboratory Medicine, Center for Neurodegenerative Disease Research, Perelman School of Medicine, Philadelphia, Pennsylvania, USA; 76 Pathology, University of Texas Southwestern Medical Center, Dallas, Texas, USA; 77 Department of Neurology, University of Chicago Medicine, Chicago, Illinois, USA; 78 Alzheimer’s Disease and Other Cognitive Disorders Unit, Neurology Service, Hospital Clínic, Institut d’Investigacions Biomèdiques August Pi i Sunyer (IDIBAPS), Universitat de Barcelona, Barcelona, Spain; 79 Neurological Tissue Bank, Biobanc-Hospital Clínic-IDIBAPS, Barcelona, Spain; 80 Servicio de Neurología, Hospital Ramón y Cajal de Madrid, Madrid, Spain; 81 Institute of Human Genetics, JLU-Gießen, Giessen, Germany; 82 Zentrum für Systemische Neurowissenschaften, Hannover, Germany

**Keywords:** multiple system atrophy, genome-wide association study, autopsy-confirmed, *ZIC1*, *ZIC4*

## Abstract

**Background:**

Multiple System Atrophy is a rare neurodegenerative disease with alpha-synuclein aggregation in glial cytoplasmic inclusions and either predominant olivopontocerebellar atrophy or striatonigral degeneration, leading to dysautonomia, parkinsonism, and cerebellar ataxia. One prior genome-wide association study in mainly clinically diagnosed patients with Multiple System Atrophy failed to identify genetic variants predisposing for the disease.

**Objective:**

Since the clinical diagnosis of Multiple System Atrophy yields a high rate of misdiagnosis when compared to the neuropathological gold standard, we studied only autopsy-confirmed cases.

**Methods:**

We studied common genetic variations in Multiple System Atrophy cases (N = 731) and controls (N = 2898).

**Results:**

The most strongly disease-associated markers were rs16859966 on chromosome 3, rs7013955 on chromosome 8, and rs116607983 on chromosome 4 with *P*-values below 5 × 10^−6^, all of which were supported by at least one additional genotyped and several imputed single nucleotide polymorphisms. The genes closest to the chromosome 3 locus are *ZIC1* and *ZIC4* encoding the zinc finger proteins of cerebellum 1 and 4 (ZIC1 and ZIC4).

**Interpretation:**

Since mutations of *ZIC1* and *ZIC4* and paraneoplastic autoantibodies directed against ZIC4 are associated with severe cerebellar dysfunction, we conducted immunohistochemical analyses in brain tissue of the frontal cortex and the cerebellum from 24 Multiple System Atrophy patients. Strong immunohistochemical expression of ZIC4 was detected in a subset of neurons of the dentate nucleus in all healthy controls and in patients with striatonigral degeneration, whereas ZIC4-immunoreactive neurons were significantly reduced inpatients with olivopontocerebellar atrophy. These findings point to a potential ZIC4-mediated vulnerability of neurons in Multiple System Atrophy.

Multiple system atrophy (MSA) is a rapidly progressive rare neurodegenerative disease presenting with variable combinations of dysautonomia, parkinsonism, and cerebellar ataxia.^1^ Two forms of MSA can be clinically distinguished, characterized by either predominant parkinsonism or predominant cerebellar symptoms.^2^ Its estimated prevalence is 3.4–4.9 cases per 100,000 individuals in the general population, and 7.8 cases per 100,000 in persons older than 40 years.^3^ The mean survival time from disease onset is 6–10 years.^4,5^ Currently, only limited symptomatic treatments and no disease-modifying therapies are available.^6^

The typical symptoms of MSA are caused by the progressive degeneration of neurons in different brain regions, particularly in the substantia nigra, striatum, inferior olivary nucleus, pons, and cerebellum, but also other parts of the central nervous systems, emphasizing the multisystem character of MSA.^2,7^ The histological hallmarks in brains of patients with MSA are glial cytoplasmic inclusions (Papp–Lantos bodies) in oligodendrocytes containing aggregated and misfolded α-synuclein.^8^ Neuropathologically, two subtypes can be distinguished, one with predominant olivopontocerebellar atrophy (OPCA), the other with mainly striatonigral degeneration (SND).^9,10^ In addition, a mixed phenotype displaying features of both OPCA and SND is found in the brains of some patients.^9,10^

The pathogenesis of MSA is unclear. MSA is considered a sporadic disease.^11^ Epidemiological studies have investigated the influence of environmental factors in MSA, including exposure to farming-related factors (pesticides, solvents, mycotoxins, dust, fuels, oils, fertilizers, animals) and certain lifestyles (consumption of well water, rural living, diet, and physical activity).^12–14^ Apart from a marginal effect of pesticides, no other environmental factors have been convincingly associated with an increased risk for development of MSA.^12−14^

Hypothesis-driven candidate gene studies have been inconsistent with respect to variants that might be associated with MSA. Associations of MSA with the genes *COQ2*, *SNCA*, *MAPT*, and *PRNP* have been discussed.^15–20^ One prior genome-wide association study (GWAS) did not identify hits of statistical significance at a genome-wide level, despite the analysis of 918 cases and 3864 controls.^21^ This GWAS had mainly included clinically diagnosed MSA cases. It needs to be stressed that clinical diagnosis is frequently not accurate in MSA. For example, a recent clinicopathological study demonstrated a false-positive diagnosis at autopsy in 38% of patients with clinically diagnosed MSA.^22^

To avoid inclusion of misdiagnosed patients in the GWAS described in this study, we included only autopsy-confirmed cases and appropriate ethnicity-matched controls.

## Subjects and Methods

### Patient Recruitment

Ethical approval had been obtained from all responsible ethics committees. All participants had given written consent.

Neuropathologists at each recruitment site (Table 1) based the definite neuropathological diagnosis of MSA on histopathological criteria, taking into account glial cytoplasmic inclusions immunoreactive for α-synuclein in characteristic anatomical distribution as a defining feature of MSA.^23^ Age, sex, disease history (including disease onset and duration), and neuropathological findings were recorded in a standardized manner for all cases.

Controls were ethnically matched to cases and either derived from biobanks KORA-gen^24^ or popGen^25^ (Europe sites) or from a North American site (Alzheimer’s Disease Genetics Consortium).^26^ The Alzheimer’s Disease Genetics Consortium assembled and genotyped DNA from subjects enrolled in the 29 NIA-Alzheimer’s Disease Centers located across the United States. For this study, the Alzheimer’s Disease Genetics Consortium provided a subset of mostly clinical, cognitively normal controls. Patients and controls were of North-Western European and African American ancestry.

### DNA Extraction

We isolated DNA from 30 mg frozen cerebellar cortex using QIAamp DNA Mini Kit (Qiagen, Venlo, the Netherlands). DNA extraction was performed at German Center for Neurodegenerative Diseases (DZNE, Munich, Germany). DNA was stored at −80°C until use. DNA concentration was measured using a NanoDrop Spectro-photometer. DNA quality was determined by gel electrophoresis.

### Genotyping

All samples were genotyped on Infinium Global Screening Arrays (Illumina, San Diego, CA, USA). The cases were genotyped at the Institute of Clinical Molecular Biology, Kiel University, Germany. The samples were genotyped in one batch on array version 2.0 for cases and version 1.0 for controls. Genotypes were called using Illumina Genome studio according to the manufacturer’s instructions using in-house cluster files.

### Quality Control and Imputation

We used PLINK (v. 1.9) [1] and R (v. 3.6.3)^27^ for all analyses. Only variants successfully genotyped in both the patient and the control populations were included in the subsequent analyses. Variants with multicharacter allele codes, insertions, deletions, duplicated markers, and all A/T and G/C variants were excluded. We excluded all samples discordant between reported and genotypic sex. Missing sex was imputed, and samples with ambiguously imputed sex were discarded. After a first step of filtering out samples and variants with call rate of less than 85%, we excluded variants with an individual call rate of less than 98% in a second filtering step. Next, we removed variants with a minor allele frequency <0.01, a significant deviation from Hardy–Weinberg equilibrium (*P* < 1 × 10^−6^) in controls, or informative missingness (*P* < 1 × 10^−5^). Subsequently, we excluded individuals with a variant call rate of <98% or an outlying heterozygosity rate (mean ± 3 standard deviations). We used a pruned dataset containing only markers in low linkage-disequilibrium regions (pairwise *r*^2^ < 0.2) to test for duplicated individuals and cryptic relatedness (Pihat > 0.125) using pairwise genome-wide estimates of the proportion of identity by descent. For each detected sample pair we excluded the individual with a lower call rate. Ethnical outliers were identified by a principal-component analysis (PCA) together with the publically available 1000 Genomes data with known ethnicities.^28^ Because the study population has genetically a mainly European ancestry, as ascertained by the PCA, we determined a European center and excluded samples more than 1.5 times the maximal European Euclidean distance away from this center. After a first association analysis of genotyped single nucleotide polymorphisms (SNPs) only, we inspected visually the cluster plots of all variants with a *P* value <1 × 10^−5^ and discarded variants without adequate cluster separation. Imputation was carried out on the quality-assured dataset using the TOPMed Imputation Server, which employs Eagle2 for phasing and minimac4 for the imputation of genotypes.^29,30^ The most likely genotype is used in downstream analyses. Variants were again filtered for minor allele frequency and deviation from Hardy–Weinberg equilibrium in controls with the same thresholds as before. In addition, SNPs with an imputation quality score *R*^2^ < 0.7 were excluded, leaving 8,131,900 variants for analyses. As a final step of the quality-control procedure, we used the R package PCAmatchR to ethnically match cases to controls with a 1:4 ratio to overcome possible difficulties with population stratification, leading to 3240 individuals for the analyses.^31^

### Association Analysis

We used logistic regression to test the additive genetic model of each marker for association with disease status. Following scree plot analysis, we incorporated the first two dimensions of the PCA and sex as covariates. We used a genome-wide significance threshold of *P* < 5 × 10^−8^ and the threshold of *P* < 5 × 10^−6^ for suggestive association. Conditional analyses, including, in turn, each SNP with a suggestive association as additional covariate, were conducted to identify adjacent independent signals. Furthermore, we tested for clumps of correlated SNPs, ie, to assess how many independent loci had been associated, and determined the number of variants supporting the lead SNP at each locus, ie, variants with P values less than the clumping threshold of 5 × 10^−5^ are in linkage disequilibrium (*r*^2^ ≥ 0.4) and not farther than 250 kb away from the respective SNP. Visualization of the results was carried out with R and LocusZoom^32^ for regional plots. Variant positions in this article are reported on human genome version 38 (GRCh38/hg38).

### Immunohistochemistry on MSA Patients’ Brain

Formalin-fixed and paraffin-embedded (FFPE) tissues from patients with MSA and controls without neurological or psychiatric diseases were obtained from the Neurobiobank Munich (Germany). All autopsy cases of the Neurobiobank Munich were collected on the basis of an informed consent according to the guidelines of the ethics commission of the Ludwig-Maximilians-University (Munich, Germany; #345–13). MSA cases had been diagnosed according to established histopathological diagnostic criteria.^10,23^

For ZIC4 immunohistochemistry, 5-μm-thick sections of FFPE tissues of the frontal cortex and the cerebellar hemisphere, including the dentate nucleus, were prepared. After deparaffinization, heat-induced epitope retrieval was performed in Tris/EDTA, pH 9, at 95°C for 30 minutes. For blocking of endogenous peroxidase and unspecific protein binding, the sections were incubated with 5% H_2_O_2_ in methanol for 20 minutes and I-Block reagent (Applied Biosystems, Waltham, MA, USA) for 15 minutes. Subsequently, ZIC4 primary antibody (rabbit, polyclonal; Merck/Sigma-Aldrich, Darmstadt, Germany) was applied overnight at 4°C at a dilution of 1:100. Signal detection was performed using the DCS *Chromo*Line DAB kit (DCS, Hamburg, Germany) according to the manufacturer’s instructions. Sections were counterstained for 1 minute with Mayer’s hemalum solution (Waldeck, Münster, Germany).

To determine the fractions of ZIC4-positive neurons of all neurons in the dentate nucleus, we scanned stained slides using a slide scanner (Axio Scan. Z1; Zeiss, Oberkochen, Germany) and visualized using the free ZEN lite software (v. 3.3; Zeiss). For statistical evaluation of the data, Student *t* test was used, and statistical significance was defined as *P* < 0.05.

## Results

### Patient Sample

**FIG. 1.**
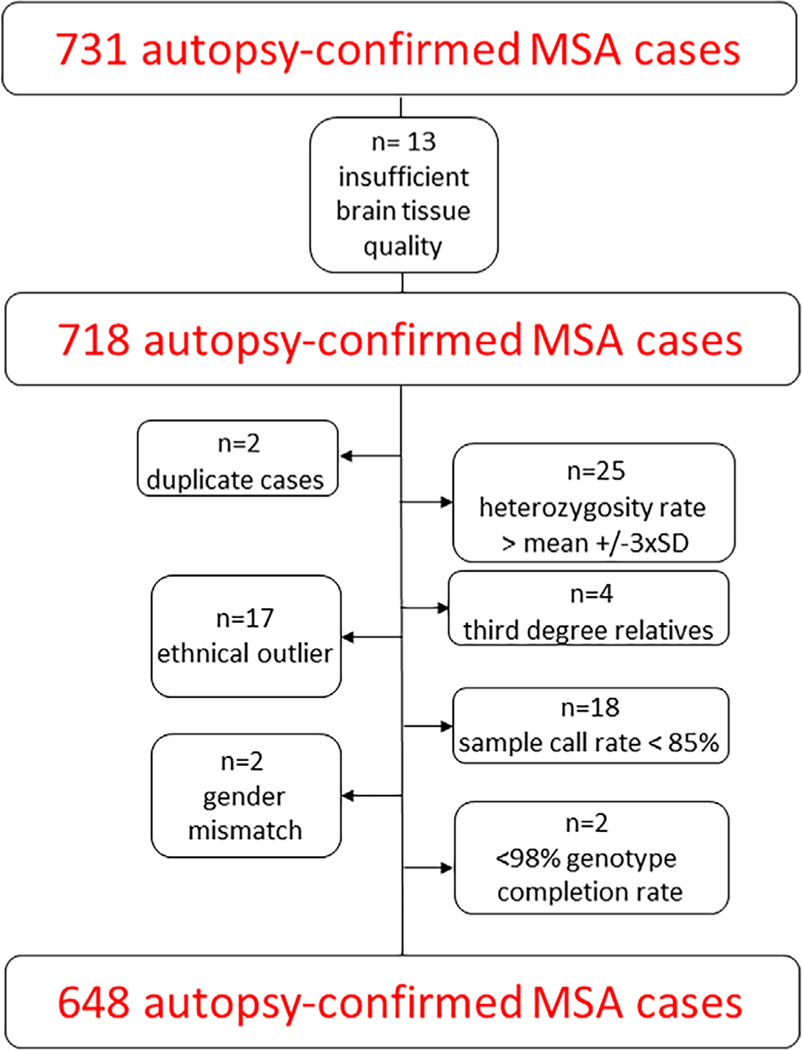
Flowchart sample quality control. SD, standard deviation. [Color figure can be viewed at wileyonlinelibrary.com]

From the initial sample of 731 cases, 13 cases had to be excluded because of insufficient tissue quality. After thorough quality control and filtering, 648 cases and 2592 controls covering 8,131,900 variants were included in the association analysis (Fig. 1). The number of excluded samples and variants in each step of the quality-control procedure is shown in Tables S1 and S2.

### Association Results

**FIG. 2.**
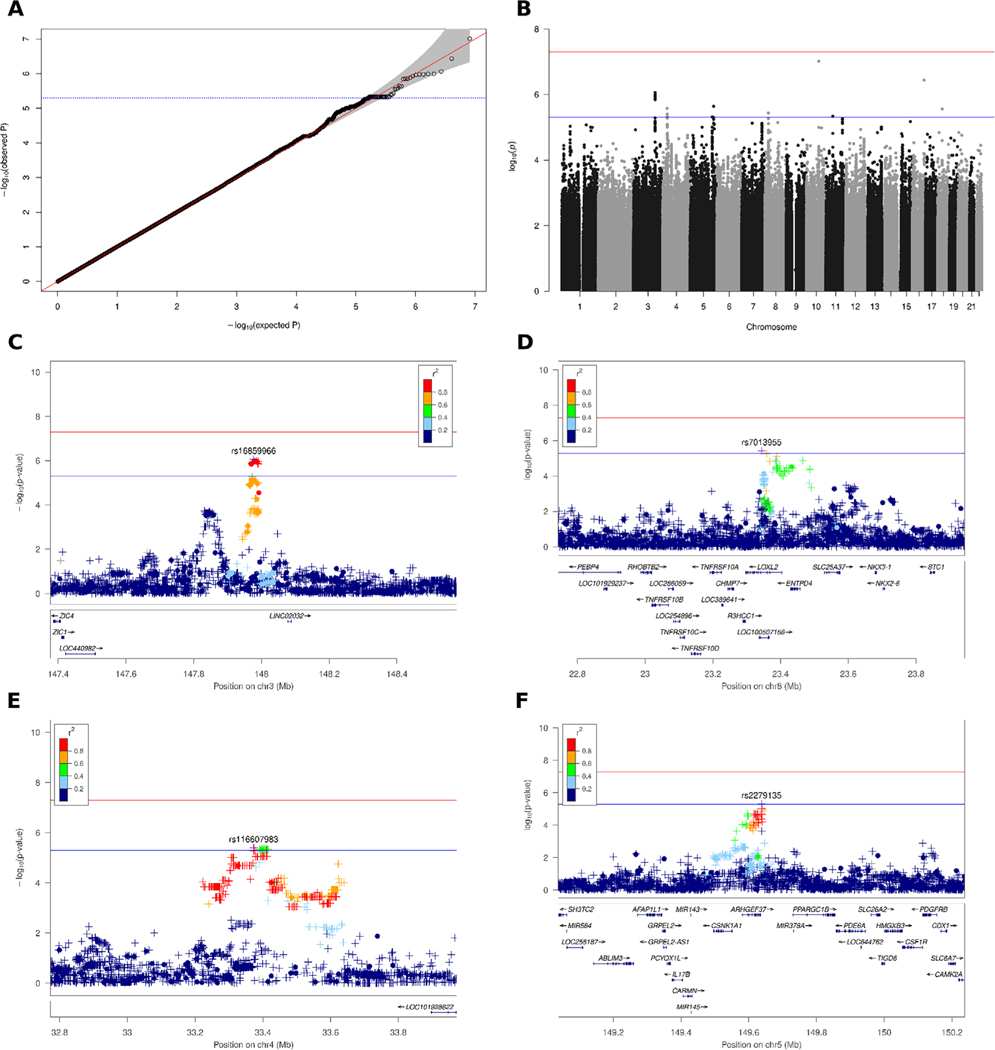
Association plots for multiple system atrophy (MSA). (**A**) QQ (quantile-quantile) plot based on 8,109,760 variants after imputation. (**B**) Manhattan plot showing –log10 *P* values from logistic regression on imputed variants with sex and two principal components as covariates plotted against their chromosomal position. The red and blue lines indicate the genome-wide significance threshold of 5 × 10^–8^ and threshold for suggestive associations of 5 × 10^–6^, respectively. (**C**) Regional plot for the association between MSA and variants on chromosome 3 in the genomic region from 147.4 to 148.6 Mb. A circle represents a genotyped variant and a plus symbol an imputed variant. The *r*^2^ metric displays the pairwise linkage-disequilibrium (LD) between the leading and the respective variant. The bottom part shows gene positions. (**D**) Regional plot for associations on chromosome 8 in the genomic region from 22.7 to 23.9 Mb. (**E**) Regional plot for associations on chromosome 4 in the genomic region from 32.8 to 34.0 Mb. (**F**) Regional plot for associations on chromosome 5 in the genomic region from 149.0 to 150.2 Mb. [Color figure can be viewed at wileyonlinelibrary.com]

We performed logistic regression incorporating sex and determined the first two dimensions of PCA as covariates using the scree plot method. The genomic inflation factor of λ = 1.01 (unimputed λ = 1.01; Fig. S1) indicates that no significant population stratification was present (Fig. 2A). We did not identify any disease-associated variants with a *P* value less than the genome-wide significance threshold of *P* < 5 × 10^−8^, but suggestive associations with *P* < 5 × 10^−6^ at 10 different loci (Fig. 2B) with the leading SNP at each locus shown in Table 2. Conditional analyses, including, in turn, any SNP with *P* < 5 × 10^−6^, excluded the presence of multiple independent signals at each locus. All variants with suggestive associations are listed in Table S3. The most noteworthy hits were rs16859966 on chromosome 3 (*P* = 8.6 × 10^−7^; odds ratio [OR], 1.58; 95% confidence interval [CI]: 1.32–1.89), rs7013955 on chromosome 8 (*P* = 3.7 × 10^−6^; OR, 1.8; 95% CI: 1.40–2.31), and rs116607983 on chromosome 4 (*P* = 4.0 × 10^−6^; OR, 2.93; 95% CI: 1.86–4.63), which were supported by at least one additional genotype, as well as several imputed SNPs with P values less than the clumping threshold of *5* × 10^−5^ as discovered in the clumping analysis (Table 2). The genes closest to the chromosome 3 locus are the Long Intergenic Non-Protein Coding RNA 2032 (*LINC02032*) approximately 100 kb downstream and the zinc-finger proteins of cerebellum 1 and 4 genes (*ZIC1, ZIC4*), located roughly 600 kb upstream (Fig. 2E). The top SNP rs7013955 on chromosome 8 maps to the lysyl oxidaselike 2 gene (*LOXL2*; Fig. 2D). The association signal around SNP rs116607983 on chromosome 4 is located in a region devoid of protein-coding genes approximately 2000 kb to either side (Fig. 2E). A fourth locus on chromosome 5 (rs2279135) was also supported by multiple clumped SNPs, but all SNPs, including the lead SNP, were imputed (Table 2). Several variants clumped at the chromosome 5 locus were located in the *ARHGEF37* gene, coding for Rho Guanine Nucleotide Exchange Factor 37 (Fig. 2F). None of the identified SNPs is an expression quantitative trait locus in brain tissues according the Genotype Tissue Expression project.^33^ At four of the six remaining loci with variants exhibiting suggestive associations, at most two supporting SNPs were present, which were all imputed; in the other two loci, no supporting SNPs could be found in the clumping analysis (Table 2, Fig. S2). We did not investigate these loci further because it is unlikely that they represent valid associations. No significant associations with Bonferroni-adjusted *P* values were detected with previously reported Parkinson’s disease associations from a meta-analysis of 17 datasets from a Parkinson’s disease GWAS (Table S4).^34^

### ZIC4 Immunohistochemistry on MSA Patients’ Brain

*ZIC4* and *ZIC1* are known to play a critical role in the embryonal development of the cerebellum. Heterozygous deletions comprising the *ZIC1* and *ZIC4* locus have been associated with the Dandy–Walker malformation, a rare congenital condition characterized by a hypoplastic cerebellar vermis and an enlarged fourth ventricle.^35,36^ In mice, deletions of *ZIC1* and *ZIC4* lead to a striking phenotype similar to the Dandy–Walker malformation with cerebellar hypoplasia and foliation defects.^35,36^ In addition, paraneoplastic autoantibodies against ZIC4 protein are linked to severe cerebellar dys-function and degeneration.^37,38^

Because cerebellar degeneration and corresponding symptoms are also a central hallmark of MSA, we decided to follow up on a potential role of ZIC4 in MSA patient brains by performing immunohistochemical stainings. For ZIC1, no primary antibody was appropriately sensitive and specific on human tissue in our hands. Thus, FFPE tissues of the cerebellum and, for comparison, the frontal cortex of patients with MSA (n = 10 SND, n = 14 OPCA/mixed phenotype) and healthy controls (b = 5) were stained with antibodies raised against ZIC4.

**FIG. 3.**
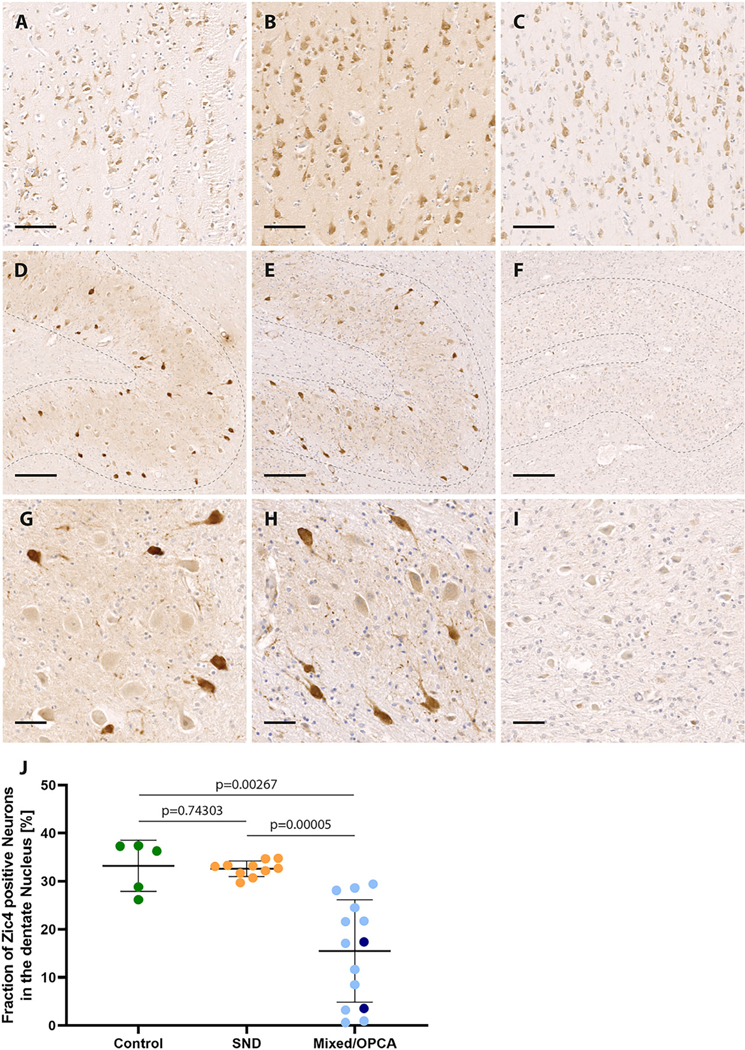
ZIC4 immunohistochemical staining of multiple system atrophy (MSA) patients and control brains. Representative ZIC4 immunohistochemical stainings of different brain regions (antibodies binding specifically to antigens in biological tissues, eg, brain tissue) of a control without neurodegenerative disease (**A, D, G**) and two MSA patients with striatonigral degeneration (SND) (**B, E, H**) and mixed subtype (**C, F, I**), respectively. (**A–C**) Nuclear and cytoplasmic expression of ZIC4 (brown staining) was detected in a comparable manner in the frontal cortex of healthy controls and patients with MSA. In the cerebellar dentate nucleus (dotted lines in D–I) of healthy controls and patients with SND, a constant subset of neurons stained strongly positive for ZIC4, whereas in patients with olivopontocerebellar atrophy (OPCA) or mixed subtype, only weak staining could be observed, and the number of ZIC4-positive neurons was clearly reduced **(D–I**, with higher magnification in **G–I)**. (**J**) Quantification of ZIC4-immunoreactive neurons in relation to the total number of neurons of the dentate nucleus depicted on the entire slide showed significantly reduced fractions of ZIC4-immunoreactive neurons in patients with either mixed subtype (light blue) or OPCA (dark blue) compared with SND or controls without neurodegenerative disease, while no difference was seen between patients with SND and healthy controls. Scale bars: 100 μm (A–C), 200 μm (D–F), 50 μm (G–I). [Color figure can be viewed at wileyonlinelibrary.com]

Nuclear and cytoplasmic staining of frontal cortex neurons was observed in all brains examined without differences between healthy controls and patients with MSA (Fig. 3A–C). In the cerebellar dentate nucleus, we found strong expression of ZIC4 in a subset of neurons in healthy controls, as well as patients with MSA with predominant SND (Fig. 3D,E,G,H). In contrast, patients with MSA with mixed subtype or OPCA showed reduced numbers of ZIC4-positive neurons, which were furthermore only weakly stained (Fig. 3F,I). Quantification of the proportions of ZIC4-positive neurons among the total number of dentate nucleus neurons depicted relatively constant proportions in healthy controls and patients with MSA-SND (33.2% ± 0.0% vs 32.6% 0.0%), whereas in patients with MSA-OPCA or MSA-mixed phenotype, we found significantly lower percentages of ZIC4-positive neurons (15.5% ± 0.1%) (Fig. 3J).

## Discussion

As part of the study, brain banks were contacted worldwide, and all available white MSA brains were included. As in the prior GWAS with 918 predominantly clinically diagnosed MSA patients, our current GWAS of 648 patients with autopsy-confirmed MSA did not identify disease-associated common variants less than the genome-wide significance threshold. Previously, hypothesis-driven candidate gene studies found inconsistent results for genetic variants and genes potentially associated with MSA. An association of MSA with genetic variants in *COQ2, SNCA, MAPT*, and *PRNP* had been discussed.^16–20,39^ However, these genes have not been convincingly confirmed in other candidate gene studies and have not been associated in a previous MSA GWAS.^21^ This preceding GWAS analyzed 918 mostly clinical cases and 3864 controls. Overall, this GWAS did not identify any genome-wide significant hits. Because our prior GWAS of 219 patients with autopsy-confirmed corticobasal degeneration did identify significant disease-associated common variants, our current findings strongly suggest that the genetic contribution to disease risk is smaller in MSA.^40^

Nevertheless, our study demonstrates several suggestive associations at different loci, which may provide relevant hypotheses for follow-up investigations into the pathogenesis of MSA.

Specifically, we identified a variant on chromosome 3 (rs16859966; *P* = 8.6 × 10^−7^; OR, 1.58; 95% CI: 1.32–1.89) located upstream of *ZIC1* and *ZIC4*. *ZIC1* and *ZIC4* are located in close genomic proximity to each other and encode transcription factors highly expressed in different brain areas.^41,42^

Proper function of these proteins is critical for the development of the CNS, particularly the cerebellum.^36^ Although no effect of rs16859966 on *ZIC1* or *ZIC4* expression is recorded in the Genotype Tissue Expression database, rare genetic variants or deletions in *ZIC1* or *ZIC4* result in congenital cerebellar defects.^35,36,43^ A heterozygous deletion of *ZIC1* and ZIC4 causes the Dandy–Walker malformation, a developmental disorder of the cerebellum.^35,44^ Remarkably, two recent epigenomic analyses in brain tissue of MSA point to *ZIC4*.^45,46^ Moreover, paraneoplastic autoantibodies against *ZIC4* induce cerebellar degeneration.^38^ Due to the pronounced cerebellar degeneration in MSA, we followed up on a possible role of ZIC4 In MSA.

Although we could detect a relatively constant proportion of approximately one-third ZIC4-positive neurons among all neurons in the cerebellar dentate nucleus in healthy controls and patients with MSASND, cases with MSA-OPCA or the mixed MSA phenotype showed significantly lower fractions of ZIC4-positive neurons. This finding suggests that ZIC4 may be involved in the neurodegeneration in MSA. The involvement of *ZIC4* mutations in the Dandy–Walker cerebellar malformation and the paraneoplastic ZIC4 autoantibody–associated cerebellar degeneration could suggest a pathomechanism in MSA, by which altered *ZIC4* expression could increase neuronal vulnerability. Further analyses of a potential functional interaction of α-synuclein and ZIC4 are currently ongoing.

Explorative analysis of PD-related associations identified by GWAS yielded no significant association in MSA when adjusting for multiple testing. However, for unadjusted *P* values, five SNPs reached a significance threshold of *P*< 0.05,which might be interesting to study further.

This study has a major limitation. Typically, a GWAS is conceptualized as a two-stage design with a discovery stage and a replication stage and supposedly achieving “genome-wide significance” in the discovery stage. The *P* values in the replication stage should remain significant after Bonferroni correction. Due to the limited number of autopsy-confirmed MSA cases worldwide, we could not conduct a two-stage procedure, let alone a further independent replication. In view of the aforementioned diagnostic uncertainty in clinical cases, a replication in predominantly clinically diagnosed MSA cases did not seem desirable.

Therefore, we strongly encourage bringing MSA cases to autopsy and conducting a further independent replication study to confirm or refute the hypotheses provided by our study.

## Supplementary Material

fS1

fS2

tS3

tS4

tS1

tS2

## Figures and Tables

**TABLE 1:** Recruitment centers and brain bank sources

City, Country	Source	MSA cases

Zürich, Switzerland	Institute of Neuropathology, University Hospital of Zurich, Zurich, Switzerland	1
Göttingen, Germany	University Medical Center Göttingen, Department of Neurology and Paracelsus-Elena-Klinik, 34,128 Kassel, Germany	2
San Francisco, CA, USA	University of California, San Francisco, CA, USA	2
Vancouver, BC, Canada	University of British Columbia, Department of Pathology and Laboratory Medicine	2
New York, NY, USA	Mount Sinai NBTR	3
Atlanta, GA, USA	Emory University, Department of Neurology & Pathology	4
Los Angeles, CA, USA	The Human Brain and Spinal Fluid Resource Center	4
Stockholm, Sweden	Department of Neurology, Karolinska University Hospital, Stockholm, Sweden	4
Vienna, Austria	Institute of Neurology, Medical University of Vienna	6
Newcastle upon Tyne, UK	Newcastle Brain Tissue Resource, Newcastle University, Campus for Ageing and Vitality, Newcastle upon Tyne NE4 5PL, UK	7
Chicago, IL, USA	University of Chicago, Department of Neurology	8
Indiana, IN, USA	Indiana University School of Medicine	8
San Diego, CA, USA	San Diego Shiley-Marcos AD Research Center, University of California	8
Tübingen, Germany	Department of Neuropathology, University Hospital of Tübingen, Tübingen, Germany	8
Madrid, Spain	Centro de Biología Molecular “Severo Ochoa,” c/Nicolás Cabrera, 1, Universidad Autónoma de Madrid, Cantoblanco, Madrid, Spain	10
Seattle, WA, USA	Department of Pathology, University of Washington, Seattle, WA, USA	10
Prague, Czech Republic	Department of Pathology and Molecular Medicine, Thomayer University Hospital, Prague	12
Sydney, NSW, Australia	Brain and Mind Centre, Sydney Medical School, The University of Sydney, Sydney, NSW, Australia	12
Arizona, AZ, USA	Banner Sun Health Research Institute	13
Parkville, VIC, Australia	Australian Brain Bank Network, Howard Florey Laboratories, The Florey Institute of Neuroscience and Mental Health	13
Dallas, TX, USA	Alzheimer’s Disease Center, University of Texas Southwestern Medical Center, Dallas, Texas, USA	15
Rosthern, SK, Canada	Saskatoon Health Region/University of Saskatchewan, Rosthern; and Movement Disorders	17
Paris, France	Raymond Escourolle Neuropathology Department, Groupe Hospitalier Pitié-Salpêtrière, Paris, France	20
London, UK	Imperial College London	22
Baltimore, MD, USA	Johns Hopkins Medical Institution Brain Resource Center, MD, USA	24
London, UK	MRC London Neurodegenerative Diseases Brain Bank, Institute of Psychiatry, Psychology and Neuroscience, King’s College	26
Munich, Germany	Neurobiobank Munich, Center for Neuropathology and Prion Research, Ludwig-Maximilians University	29
Boston, MA, USA	Massachusetts General Hospital	30
Barcelona, Spain	Neurological Tissue Bank of the Biobanc-Hospital Clinic-IDIBAPS	34
Amsterdam, the Netherlands	Alzheimer Center	36
Ann Arbor, MI, USA	University of Michigan, Department of Pathology, University of Michigan Medical School, Ann Arbor, MI, USA	37
Miami, FL, USA	UM Brain Endowment Bank, an NIH NeuroBioBank	45
Philadelphia, PA, USA	The Penn FTD Center — University of Pennsylvania, USA	54
Jacksonville, FL, USA	Department of Neuroscience, Mayo Clinic, Jacksonville	205
**Total**		**731**

MSA, multiple system atrophy.

**TABLE 2:** Top SNPs at each locus with P < 5 × 10^−6^

				MAF					No. of SNPs in Clump		
								
CHR	dbSNP ID	BP	Minor allele	Cases	Controls	OR (95% CI)	*P*	IM/GT	Total	GT	IM

10	rs4933352	85,280,795	G	0.42	0.52	0.71 (0.62–0.80)	9.7E-08	IM	2	0	2
16	rs79418449	80,515,374	C	0.04	0.02	2.54 (1.77–3.63)	3.7E-07	GT	1	1	0
3	rs16859966	147,976,678	G	0.17	0.12	1.58 (1.32–1.89)	8.6E-07	IM	45	24	21
5	rs114019803	159,559,041	T	0.02	0.01	3.36 (2.03–5.56)	2.3E-06	IM	3	0	3
4	rs933953	31,356,173	C	0.25	0.32	0.71 (0.62–0.82)	2.6E-06	IM	1	0	1
18	rs116914137	30,589,500	A	0.05	0.02	2.17 (1.57–3.00)	2.8E-06	IM	3	0	3
8	rs7013955	23,343,590	A	0.08	0.05	1.80 (1.40–2.31)	3.7E-06	IM	20	1	19
4	rs116607983	33,372,461	A	0.03	0.01	2.93 (1.86–4.63)	4.0E-06	IM	88	3	85
11	rs141819348	47,698,235	T	0.05	0.03	2.10 (1.53–2.88)	4.6E-06	IM	3	0	3
5	rs2279135	149,637,742	C	0.32	0.27	1.39 (1.21–1.60)	4.8E-06	IM	24	0	24

Results from an association analysis with logistic regression including sex and the first two dimensions of principal-component analysis (PCA) as covariates in 648 cases with MSA and 2898 controls. For an OR < 1, the minor allele has a protective effect, whereas an OR > 1 indicates that the minor allele is associated with an increased risk for development of the disease. Only the leading SNP at each locus with a suggestive association between the disease status and a variant is reported. Table S3 lists all suggestive associations.

SNP, single-nucleotide polymorphism; CHR, chromosome; dbSNP, database of single-nucleotide polymorphism; BP, base-pair coordinates according to human reference genome GRCh38; MAF, minor allele frequency; OR, odds ratio; CI, confidence interval; IM, imputed; GT, genotyped.

## Data Availability

The data that support the findings of this study are available from the corresponding author upon reasonable request.
