# Supplementary figures and images for: Common Variants Near ZIC1 and ZIC4 in Autopsy-Confirmed Multiple System Atrophy
Source: Mov Disord. Author manuscript; Available in PMC 2023 Oct 1. (PMC10052809; doi:10.1002/mds.29164)

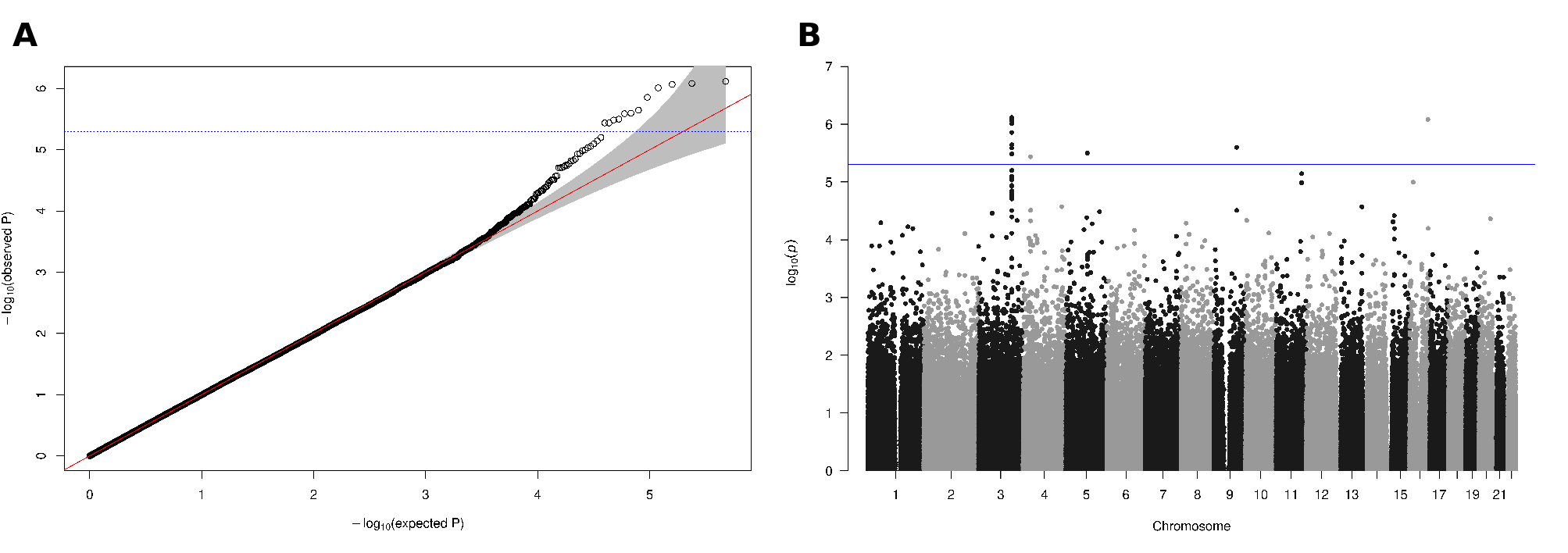

Supplement: fS1 [file NIHMS1869649-supplement-fS1.tiff]

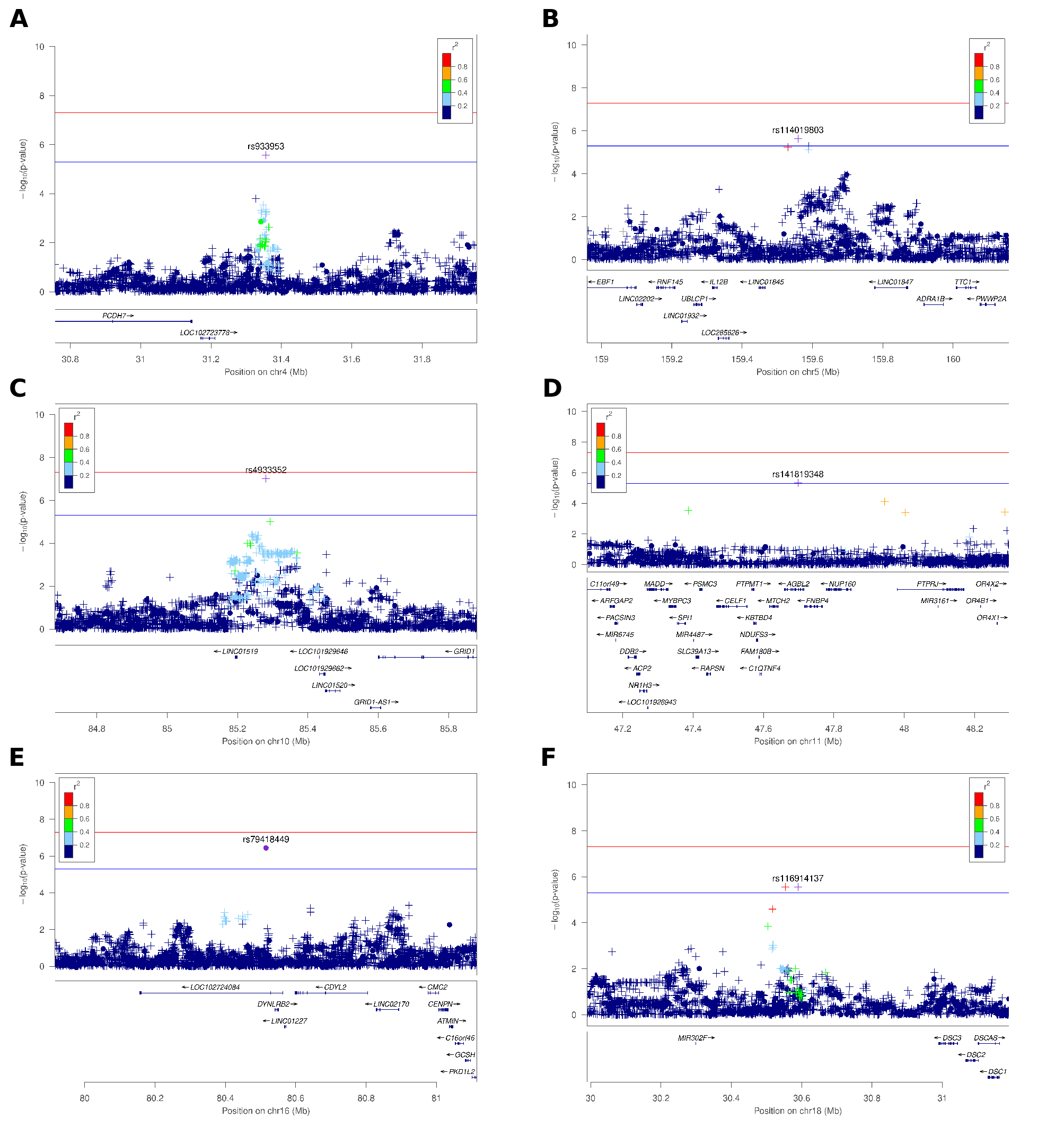

Supplement: fS2 [file NIHMS1869649-supplement-fS2.tiff]
