# Supplementary material for: Common Variants Near ZIC1 and ZIC4 in Autopsy-Confirmed Multiple System Atrophy
Source: Mov Disord. Author manuscript; Available in PMC 2023 Oct 1. (PMC10052809; doi:10.1002/mds.29164)
Supplement: tS3 [file NIHMS1869649-supplement-tS3.docx]

**Supplementary material**

**Supplementary Table S3: Identified associations with P < 5 x 10^-6^.**

Results from association analysis with logistic regression including sex and the first two dimensions of PCA as covariates in 648 cases with MSA and 2,898 controls. For an OR < 1, the minor allele has a protective effect whereas an OR > 1 indicates that the minor allele is associated with an increased risk of developing the disease. BP = base-pair coordinates according to human reference genome GRCh38, CHR = Chromosome, dbSNP = database of single nucleotide polymorphisms, GT = genotyped, IM = imputed, L95 = Lower bound of 95% confidence interval for odds ratio, MAF = minor allele frequency, U95= Upper bound of 95% confidence interval for odds ratio.

| CHR | dbSNP ID | BP |  | MAF | | | OR | L95 | U95 | P | IM/ GT |
| --- | --- | --- | --- | --- | --- | --- | --- | --- | --- | --- | --- |
|  |  |  | Minor allele | Cases | Controls | All |  |  |  |  |  |
| **10** | **rs4933352** | **85280795** | **G** | **0.42** | **0.52** | **0.50** | **0.71** | **0.62** | **0.80** | **9.7E-08** | **IM** |
| **16** | **rs79418449** | **80515374** | **C** | **0.04** | **0.02** | **0.03** | **2.54** | **1.77** | **3.63** | **3.7E-07** | **GT** |
| **3** | **rs16859966** | **147976678** | **G** | **0.17** | **0.12** | **0.13** | **1.58** | **1.32** | **1.89** | **8.6E-07** | **IM** |
| 3 | rs80100284 | 147978148 | A | 0.16 | 0.12 | 0.13 | 1.57 | 1.31 | 1.89 | 1E-06 | GT |
| 3 | rs17528799 | 147980674 | C | 0.16 | 0.12 | 0.13 | 1.57 | 1.31 | 1.89 | 1E-06 | GT |
| 3 | rs79713750 | 147985042 | C | 0.16 | 0.12 | 0.13 | 1.57 | 1.31 | 1.89 | 1E-06 | GT |
| 3 | rs17529042 | 147985655 | C | 0.16 | 0.12 | 0.13 | 1.57 | 1.31 | 1.89 | 1E-06 | IM |
| 3 | rs6787937 | 147991156 | C | 0.16 | 0.12 | 0.13 | 1.57 | 1.31 | 1.89 | 1.1E-06 | IM |
| 3 | rs17588473 | 147987565 | G | 0.16 | 0.12 | 0.13 | 1.57 | 1.31 | 1.88 | 1.2E-06 | GT |
| 3 | rs149669221 | 147988874 | A | 0.16 | 0.12 | 0.13 | 1.57 | 1.31 | 1.88 | 1.3E-06 | IM |
| 3 | rs62277129 | 147968720 | C | 0.16 | 0.12 | 0.12 | 1.57 | 1.31 | 1.88 | 1.4E-06 | GT |
| 3 | rs62273877 | 147990478 | G | 0.16 | 0.12 | 0.13 | 1.57 | 1.31 | 1.88 | 1.4E-06 | IM |
| 3 | rs115055496 | 147970642 | G | 0.16 | 0.12 | 0.13 | 1.57 | 1.31 | 1.88 | 1.4E-06 | GT |
| **5** | **rs114019803** | **159559041** | **T** | **0.02** | **0.01** | **0.01** | **3.36** | **2.03** | **5.56** | **2.3E-06** | **IM** |
| 5 | rs148637949 | 159559783 | G | 0.02 | 0.01 | 0.01 | 3.36 | 2.03 | 5.56 | 2.3E-06 | IM |
| **4** | **rs933953** | **31356173** | **C** | **0.25** | **0.32** | **0.30** | **0.71** | **0.62** | **0.82** | **2.6E-06** | **IM** |
| **18** | **rs116914137** | **30589500** | **A** | **0.05** | **0.02** | **0.03** | **2.17** | **1.57** | **3.00** | **2.8E-06** | **IM** |
| 18 | rs146733256 | 30553996 | A | 0.05 | 0.02 | 0.03 | 2.21 | 1.59 | 3.08 | 2.8E-06 | IM |
| **8** | **rs7013955** | **23343590** | **A** | **0.08** | **0.05** | **0.06** | **1.80** | **1.40** | **2.31** | **3.7E-06** | **IM** |
| **4** | **rs116607983** | **33372461** | **A** | **0.03** | **0.01** | **0.01** | **2.93** | **1.86** | **4.63** | **4E-06** | **IM** |
| 4 | rs115369416 | 33373550 | T | 0.03 | 0.01 | 0.01 | 2.93 | 1.86 | 4.63 | 4E-06 | IM |
| **11** | **rs141819348** | **47698235** | **T** | **0.05** | **0.03** | **0.03** | **2.10** | **1.53** | **2.88** | **4.6E-06** | **IM** |
| 4 | rs55681260 | 33390689 | T | 0.06 | 0.03 | 0.03 | 2.05 | 1.51 | 2.78 | 4.7E-06 | IM |
| 4 | rs1506250 | 33391458 | G | 0.06 | 0.03 | 0.03 | 2.05 | 1.51 | 2.78 | 4.7E-06 | IM |
| 4 | rs74927140 | 33392112 | G | 0.06 | 0.03 | 0.03 | 2.05 | 1.51 | 2.78 | 4.7E-06 | IM |
| 4 | rs73823107 | 33396305 | A | 0.06 | 0.03 | 0.03 | 2.05 | 1.51 | 2.78 | 4.7E-06 | IM |
| 4 | rs12647606 | 33396483 | C | 0.06 | 0.03 | 0.03 | 2.05 | 1.51 | 2.78 | 4.7E-06 | GT |
| 4 | rs12643899 | 33396765 | T | 0.06 | 0.03 | 0.03 | 2.05 | 1.51 | 2.78 | 4.7E-06 | GT |
| 4 | rs1911023 | 33397756 | T | 0.06 | 0.03 | 0.03 | 2.05 | 1.51 | 2.78 | 4.7E-06 | IM |
| 4 | rs12649232 | 33398189 | C | 0.06 | 0.03 | 0.03 | 2.05 | 1.51 | 2.78 | 4.7E-06 | IM |
| 4 | rs7658371 | 33399068 | C | 0.06 | 0.03 | 0.03 | 2.05 | 1.51 | 2.78 | 4.7E-06 | IM |
| 4 | rs76588230 | 33399670 | G | 0.06 | 0.03 | 0.03 | 2.05 | 1.51 | 2.78 | 4.7E-06 | IM |
| 4 | rs78164979 | 33402066 | A | 0.06 | 0.03 | 0.03 | 2.05 | 1.51 | 2.78 | 4.7E-06 | IM |
| 4 | rs77400205 | 33402096 | T | 0.06 | 0.03 | 0.03 | 2.05 | 1.51 | 2.78 | 4.7E-06 | IM |
| 4 | rs28758918 | 33402691 | A | 0.06 | 0.03 | 0.03 | 2.05 | 1.51 | 2.78 | 4.7E-06 | IM |
| 4 | rs73823113 | 33403579 | A | 0.06 | 0.03 | 0.03 | 2.05 | 1.51 | 2.78 | 4.7E-06 | IM |
| 4 | rs73823114 | 33403863 | G | 0.06 | 0.03 | 0.03 | 2.05 | 1.51 | 2.78 | 4.7E-06 | IM |
| 4 | rs73823115 | 33403917 | A | 0.06 | 0.03 | 0.03 | 2.05 | 1.51 | 2.78 | 4.7E-06 | IM |
| 4 | rs7657687 | 33404711 | G | 0.06 | 0.03 | 0.03 | 2.05 | 1.51 | 2.78 | 4.7E-06 | IM |
| 4 | rs35894559 | 33405305 | G | 0.06 | 0.03 | 0.03 | 2.05 | 1.51 | 2.78 | 4.7E-06 | IM |
| 4 | rs73823117 | 33408795 | A | 0.06 | 0.03 | 0.03 | 2.05 | 1.51 | 2.78 | 4.7E-06 | IM |
| 4 | rs73823119 | 33411185 | C | 0.06 | 0.03 | 0.03 | 2.05 | 1.51 | 2.78 | 4.7E-06 | IM |
| 4 | rs73823120 | 33411587 | A | 0.06 | 0.03 | 0.03 | 2.05 | 1.51 | 2.78 | 4.7E-06 | IM |
| 4 | rs79060702 | 33412974 | A | 0.06 | 0.03 | 0.03 | 2.05 | 1.51 | 2.78 | 4.7E-06 | IM |
| 4 | rs56365829 | 33413459 | A | 0.06 | 0.03 | 0.03 | 2.05 | 1.51 | 2.78 | 4.7E-06 | IM |
| 4 | rs6822915 | 33414893 | A | 0.06 | 0.03 | 0.03 | 2.05 | 1.51 | 2.78 | 4.7E-06 | IM |
| 4 | rs12641918 | 33415336 | G | 0.06 | 0.03 | 0.03 | 2.05 | 1.51 | 2.78 | 4.7E-06 | IM |
| 4 | rs12642719 | 33415558 | C | 0.06 | 0.03 | 0.03 | 2.05 | 1.51 | 2.78 | 4.7E-06 | IM |
| **5** | **rs2279135** | **149637742** | **C** | **0.32** | **0.27** | **0.28** | **1.39** | **1.21** | **1.60** | **4.8E-06** | **IM** |
