# Supplementary material for: Common Variants Near ZIC1 and ZIC4 in Autopsy-Confirmed Multiple System Atrophy
Source: Mov Disord. Author manuscript; Available in PMC 2023 Oct 1. (PMC10052809; doi:10.1002/mds.29164)
Supplement: tS4 [file NIHMS1869649-supplement-tS4.docx]

**Supplementary Table S4: Top Parkinson's disease SNPs according to the Parkinson's disease meta-analysis of genome-wide association studies, Nalls et al. 2019.**

The SNP selection is based on results from the meta-analysis of 17 datasets from Parkinson's disease GWAS available from European ancestry samples. BP = base-pair coordinates according to human reference genome GRCh38, CHR = Chromosome, dbSNP = database of single nucleotide polymorphisms, L95 = Lower bound of 95% confidence interval for odds ratio, MAF = minor allele frequency, P adj = Bonferroni-adjusted P-values, U95= Upper bound of 95% confidence interval for odds ratio.

| CHR | dbSNP ID | BP | MAF | | | OR | L95 | U95 | P | P adj |
| --- | --- | --- | --- | --- | --- | --- | --- | --- | --- | --- |
|  |  |  | Cases | Controls | All |  |  |  |  |  |
| 1 | rs114138760 | 154925709 | 0.01 | 0.01 | 0.01 | 1.06 | 0.58 | 1.94 | 0.8446 | 1 |
| 1 | rs35749011 | 155162560 | 0.01 | 0.01 | 0.01 | 1.06 | 0.60 | 1.88 | 0.8480 | 1 |
| 1 | rs6658353 | 161499264 | 0.49 | 0.47 | 0.48 | 1.00 | 0.88 | 1.13 | 0.9814 | 1 |
| 1 | rs11578699 | 171750629 | 0.19 | 0.19 | 0.19 | 1.00 | 0.85 | 1.18 | 0.9792 | 1 |
| 1 | rs823118 | 205754444 | 0.42 | 0.42 | 0.42 | 0.97 | 0.85 | 1.10 | 0.5969 | 1 |
| 1 | rs11557080 | 205768611 | 0.15 | 0.14 | 0.14 | 1.11 | 0.93 | 1.33 | 0.2516 | 1 |
| 1 | rs4653767 | 226728377 | 0.29 | 0.28 | 0.28 | 1.03 | 0.90 | 1.19 | 0.6553 | 1 |
| 1 | rs10797576 | 232528865 | 0.14 | 0.14 | 0.14 | 0.94 | 0.79 | 1.13 | 0.5095 | 1 |
| 2 | rs76116224 | 17966582 | 0.10 | 0.10 | 0.10 | 1.13 | 0.91 | 1.39 | 0.2654 | 1 |
| 2 | rs2042477 | 95335195 | 0.24 | 0.25 | 0.25 | 0.91 | 0.78 | 1.06 | 0.2227 | 1 |
| 2 | rs11683001 | 101780501 | 0.31 | 0.31 | 0.31 | 0.90 | 0.78 | 1.03 | 0.1206 | 1 |
| 2 | rs57891859 | 134707046 | 0.29 | 0.28 | 0.28 | 1.06 | 0.92 | 1.21 | 0.4523 | 1 |
| 2 | rs1474055 | 168253884 | 0.13 | 0.13 | 0.13 | 0.98 | 0.81 | 1.18 | 0.8519 | 1 |
| 3 | rs73038319 | 18320267 | 0.04 | 0.05 | 0.05 | 1.02 | 0.74 | 1.41 | 0.9166 | 1 |
| 3 | rs6808178 | 28664199 | 0.36 | 0.36 | 0.36 | 1.00 | 0.88 | 1.14 | 0.9947 | 1 |
| 3 | rs12497850 | 48711556 | 0.36 | 0.37 | 0.37 | 0.97 | 0.85 | 1.11 | 0.6620 | 1 |
| 3 | rs55961674 | 122478045 | 0.17 | 0.17 | 0.17 | 1.01 | 0.85 | 1.20 | 0.9324 | 1 |
| 3 | rs11707416 | 151391177 | 0.37 | 0.38 | 0.38 | 0.94 | 0.82 | 1.08 | 0.3755 | 1 |
| 3 | rs1450522 | 161359842 | 0.32 | 0.32 | 0.32 | 1.00 | 0.88 | 1.15 | 0.9714 | 1 |
| 3 | rs10513789 | 183042285 | 0.20 | 0.21 | 0.21 | 0.95 | 0.82 | 1.12 | 0.5535 | 1 |
| 4 | rs873786 | 931588 | 0.10 | 0.11 | 0.11 | 0.90 | 0.73 | 1.11 | 0.3307 | 1 |
| 4 | rs34311866 | 958159 | 0.20 | 0.19 | 0.19 | 1.09 | 0.93 | 1.29 | 0.2965 | 1 |
| 4 | rs4698412 | 15735725 | 0.45 | 0.46 | 0.46 | 0.95 | 0.84 | 1.08 | 0.4439 | 1 |
| 4 | rs34025766 | 17967188 | 0.16 | 0.15 | 0.15 | 0.95 | 0.80 | 1.14 | 0.5962 | 1 |
| 4 | rs6825004 | 76189212 | 0.30 | 0.32 | 0.32 | 0.92 | 0.80 | 1.06 | 0.2461 | 1 |
| 4 | rs4101061 | 76226816 | 0.28 | 0.29 | 0.29 | 0.96 | 0.84 | 1.11 | 0.6118 | 1 |
| 4 | rs6854006 | 76276901 | 0.36 | 0.37 | 0.37 | 0.92 | 0.80 | 1.05 | 0.1909 | 1 |
| 4 | rs356182 | 89704960 | 0.33 | 0.34 | 0.34 | 0.93 | 0.81 | 1.07 | 0.3020 | 1 |
| 4 | rs5019538 | 89715479 | 0.30 | 0.30 | 0.30 | 0.98 | 0.85 | 1.12 | 0.7275 | 1 |
| 4 | rs13117519 | 113447909 | 0.17 | 0.17 | 0.17 | 1.02 | 0.86 | 1.20 | 0.8481 | 1 |
| 4 | rs62333164 | 169662006 | 0.33 | 0.34 | 0.34 | 0.96 | 0.84 | 1.10 | 0.5464 | 1 |
| 5 | rs1867598 | 60842132 | 0.10 | 0.09 | 0.09 | 1.16 | 0.94 | 1.43 | 0.1691 | 1 |
| 5 | rs26431 | 103030090 | 0.29 | 0.30 | 0.30 | 0.95 | 0.83 | 1.10 | 0.5002 | 1 |
| 5 | rs11950533 | 134863415 | 0.11 | 0.11 | 0.11 | 1.02 | 0.83 | 1.25 | 0.8480 | 1 |
| 6 | rs4140646 | 27771022 | 0.22 | 0.22 | 0.22 | 0.98 | 0.84 | 1.15 | 0.8445 | 1 |
| 6 | rs9261484 | 30140906 | 0.24 | 0.26 | 0.25 | 0.95 | 0.82 | 1.10 | 0.4728 | 1 |
| 6 | rs504594 | 32610995 | 0.17 | 0.16 | 0.16 | 1.02 | 0.86 | 1.21 | 0.8057 | 1 |
| 6 | rs12528068 | 71778059 | 0.28 | 0.29 | 0.28 | 0.96 | 0.84 | 1.11 | 0.6098 | 1 |
| 6 | rs997368 | 111922088 | 0.21 | 0.18 | 0.19 | 1.09 | 0.93 | 1.28 | 0.3007 | 1 |
| 6 | rs75859381 | 132889222 | 0.02 | 0.03 | 0.03 | 0.92 | 0.61 | 1.39 | 0.6934 | 1 |
| 7 | rs199351 | 23260430 | 0.44 | 0.40 | 0.41 | 1.24 | 1.09 | 1.42 | 0.0009 | 0.08 |
| 7 | rs76949143 | 66544864 | 0.05 | 0.04 | 0.05 | 1.14 | 0.85 | 1.52 | 0.3754 | 1 |
| 8 | rs1293298 | 11854934 | 0.26 | 0.27 | 0.27 | 0.96 | 0.83 | 1.11 | 0.5876 | 1 |
| 8 | rs620513 | 16840084 | 0.29 | 0.28 | 0.28 | 1.02 | 0.88 | 1.17 | 0.8140 | 1 |
| 8 | rs2280104 | 22668467 | 0.36 | 0.36 | 0.36 | 1.00 | 0.88 | 1.14 | 0.9926 | 1 |
| 8 | rs2086641 | 129889663 | 0.29 | 0.27 | 0.27 | 1.09 | 0.94 | 1.26 | 0.2370 | 1 |
| 9 | rs13294100 | 17579692 | 0.35 | 0.36 | 0.36 | 1.01 | 0.88 | 1.15 | 0.9203 | 1 |
| 9 | rs10756907 | 17727067 | 0.22 | 0.22 | 0.22 | 0.97 | 0.83 | 1.13 | 0.6608 | 1 |
| 9 | rs6476434 | 34046393 | 0.27 | 0.27 | 0.27 | 1.01 | 0.87 | 1.16 | 0.9169 | 1 |
| 10 | rs896435 | 15515407 | 0.33 | 0.32 | 0.33 | 1.05 | 0.92 | 1.20 | 0.4620 | 1 |
| 10 | rs10748818 | 102255522 | 0.15 | 0.15 | 0.15 | 1.04 | 0.88 | 1.24 | 0.6374 | 1 |
| 10 | rs72840788 | 119656173 | 0.20 | 0.21 | 0.21 | 1.01 | 0.87 | 1.18 | 0.8767 | 1 |
| 10 | rs117896735 | 119776815 | 0.01 | 0.02 | 0.02 | 0.69 | 0.40 | 1.21 | 0.1930 | 1 |
| 11 | rs7938782 | 10537230 | 0.14 | 0.12 | 0.12 | 1.12 | 0.93 | 1.34 | 0.2509 | 1 |
| 11 | rs12283611 | 83776234 | 0.44 | 0.42 | 0.42 | 1.08 | 0.95 | 1.23 | 0.2307 | 1 |
| 11 | rs3802920 | 133917106 | 0.20 | 0.19 | 0.19 | 1.01 | 0.86 | 1.18 | 0.9229 | 1 |
| 12 | rs76904798 | 40220632 | 0.15 | 0.14 | 0.14 | 1.13 | 0.95 | 1.35 | 0.1767 | 1 |
| 12 | rs7134559 | 46025303 | 0.41 | 0.40 | 0.40 | 0.98 | 0.86 | 1.12 | 0.8099 | 1 |
| 12 | rs10847864 | 122842051 | 0.34 | 0.34 | 0.34 | 0.97 | 0.85 | 1.11 | 0.6768 | 1 |
| 12 | rs11610045 | 132487182 | 0.48 | 0.50 | 0.50 | 0.94 | 0.82 | 1.07 | 0.3192 | 1 |
| 13 | rs9568188 | 49353596 | 0.26 | 0.27 | 0.27 | 1.01 | 0.87 | 1.16 | 0.9488 | 1 |
| 13 | rs4771268 | 97212767 | 0.23 | 0.22 | 0.22 | 1.02 | 0.88 | 1.19 | 0.7802 | 1 |
| 14 | rs12147950 | 37520065 | 0.44 | 0.44 | 0.44 | 0.95 | 0.83 | 1.08 | 0.4011 | 1 |
| 14 | rs11158026 | 54882151 | 0.35 | 0.32 | 0.32 | 1.13 | 0.98 | 1.29 | 0.0841 | 1 |
| 14 | rs3742785 | 74906331 | 0.22 | 0.21 | 0.21 | 1.06 | 0.91 | 1.24 | 0.4377 | 1 |
| 14 | rs979812 | 87997920 | 0.47 | 0.43 | 0.43 | 1.17 | 1.03 | 1.33 | 0.0195 | 1 |
| 15 | rs2251086 | 61705186 | 0.15 | 0.16 | 0.16 | 1.01 | 0.84 | 1.20 | 0.9557 | 1 |
| 16 | rs6497339 | 19266171 | 0.44 | 0.46 | 0.46 | 0.93 | 0.82 | 1.05 | 0.2523 | 1 |
| 16 | rs2904880 | 28933075 | 0.32 | 0.33 | 0.32 | 1.00 | 0.87 | 1.15 | 0.9944 | 1 |
| 16 | rs11150601 | 30966478 | 0.38 | 0.36 | 0.37 | 1.09 | 0.95 | 1.24 | 0.2190 | 1 |
| 16 | rs6500328 | 50702745 | 0.41 | 0.40 | 0.40 | 1.02 | 0.89 | 1.16 | 0.8151 | 1 |
| 16 | rs3104783 | 52602330 | 0.44 | 0.42 | 0.42 | 1.07 | 0.94 | 1.21 | 0.3111 | 1 |
| 16 | rs10221156 | 52935514 | 0.10 | 0.10 | 0.10 | 0.97 | 0.78 | 1.20 | 0.7499 | 1 |
| 17 | rs12600861 | 7452302 | 0.36 | 0.35 | 0.35 | 1.02 | 0.89 | 1.17 | 0.7553 | 1 |
| 17 | rs12951632 | 42588995 | 0.28 | 0.26 | 0.27 | 1.07 | 0.93 | 1.24 | 0.3285 | 1 |
| 17 | rs2269906 | 44216969 | 0.35 | 0.33 | 0.33 | 1.10 | 0.96 | 1.25 | 0.1761 | 1 |
| 17 | rs850738 | 44357262 | 0.39 | 0.42 | 0.41 | 0.90 | 0.79 | 1.03 | 0.1150 | 1 |
| 17 | rs62053943 | 45666837 | 0.17 | 0.15 | 0.15 | 1.09 | 0.92 | 1.29 | 0.3456 | 1 |
| 17 | rs117615688 | 45720942 | 0.07 | 0.06 | 0.06 | 0.99 | 0.78 | 1.27 | 0.9590 | 1 |
| 17 | rs11658976 | 46789439 | 0.42 | 0.40 | 0.40 | 1.13 | 0.99 | 1.29 | 0.0635 | 1 |
| 17 | rs61169879 | 61840005 | 0.18 | 0.16 | 0.17 | 1.18 | 1.00 | 1.40 | 0.0458 | 1 |
| 17 | rs666463 | 78429399 | 0.14 | 0.17 | 0.16 | 0.76 | 0.63 | 0.91 | 0.0026 | 0.22 |
| 18 | rs1941685 | 33724354 | 0.52 | 0.49 | 0.49 | 1.13 | 0.99 | 1.28 | 0.0610 | 1 |
| 18 | rs12456492 | 43093415 | 0.32 | 0.32 | 0.32 | 1.02 | 0.89 | 1.17 | 0.7305 | 1 |
| 18 | rs8087969 | 51157219 | 0.46 | 0.46 | 0.46 | 1.03 | 0.91 | 1.17 | 0.5991 | 1 |
| 19 | rs55818311 | 2341049 | 0.32 | 0.32 | 0.32 | 0.99 | 0.87 | 1.14 | 0.9333 | 1 |
| 20 | rs77351827 | 6025395 | 0.15 | 0.12 | 0.13 | 1.21 | 1.01 | 1.45 | 0.0397 | 1 |
| 21 | rs2248244 | 37480059 | 0.26 | 0.27 | 0.27 | 0.97 | 0.84 | 1.12 | 0.7098 | 1 |
