# Supplementary material for: Common Variants Near ZIC1 and ZIC4 in Autopsy-Confirmed Multiple System Atrophy
Source: Mov Disord. Author manuscript; Available in PMC 2023 Oct 1. (PMC10052809; doi:10.1002/mds.29164)
Supplement: tS1 [file NIHMS1869649-supplement-tS1.docx]

**Supplementary material**

**Supplementary Table S1**: **Summary of per sample quality control.** SD = standard deviation.

|  | **Cases** | **Controls** | **Total** |
| --- | --- | --- | --- |
| **Samples started with** | **731** | **2,898** | **3,629** |
|  | Number of excluded samples | | |
| Insufficient brain tissue quality | 13 | 0 | 13 |
| Duplicate Samples | 2 | 0 | 2 |
| Sample call rate < 85% | 18 | 0 | 18 |
| Sample Call rate < 98% | 2 | 3 | 5 |
| Heterozygosity Rate > Mean ± 3xSD | 25 | 20 | 45 |
| Cryptic relatedness > third degree relatives | 4 | 33 | 37 |
| Missing or discrepant sex | 2 | 9 | 11 |
| Ethnical Outlier | 17 | 0 | 17 |
| Control Matching | - | 241 | 241 |
| Total samples excluded | 68 | 306 | 374 |
| **Total samples analysed** | **648** | **2,592** | **3,240** |
