# Supplementary material for: Common Variants Near ZIC1 and ZIC4 in Autopsy-Confirmed Multiple System Atrophy
Source: Mov Disord. Author manuscript; Available in PMC 2023 Oct 1. (PMC10052809; doi:10.1002/mds.29164)
Supplement: tS2 [file NIHMS1869649-supplement-tS2.docx]

**Supplementary material**

**Supplementary Table S2:** **Summary of per marker quality control.** MAF = minor allele frequency, HWE = Hardy-Weinberg equilibrium.

**A**: Quality control on genotyped data

| **Markers started with** | **599,560** |
| --- | --- |
|  | Number of excluded markers |
| Reducing to autosomes | 14,407 |
| Marker Call rate < 85% | 203 |
| Marker Call rate < 98% | 5,453 |
| MAF < 0.01 | 99,500 |
| HWE in controls with P < 10E-6 | 527 |
| Informative missingness with P < 10E-5 | 2,169 |
| No cluster separation | 40 |
| Total markers excluded | 122,299 |
| **Markers used in analysis** | **477,261** |

**B**: Quality Control on imputed variants

| **Markers after Imputation** | **271,284,138** |
| --- | --- |
|  | Number of excluded markers |
| MAF < 0.01 | 263,056,018 |
| HWE in controls with P < 10E-6 | 465 |
| Informative missingness with P < 10E-5 | 117,895 |
| Total markers excluded | 263,174,378 |
| **Markers used in analysis** | **8,109,760** |
